# Recent advances in feeder-free NK cell expansion as a future direction of adoptive cell therapy

**DOI:** 10.3389/fimmu.2025.1675353

**Published:** 2025-09-29

**Authors:** Jimin Lee, Munsik Kim, Hanbin Jeong, Ilkoo Noh, Jeehun Park

**Affiliations:** ^1^ Department of Molecular Bioscience, College of Biomedical Science, Kangwon National University, Chuncheon, Republic of Korea; ^2^ Division of Biomedical Convergence, College of Biomedical Science, Kangwon National University, Chuncheon, Republic of Korea; ^3^ Multidimensional Genomics Research Center, Kangwon National University, Chuncheon, Republic of Korea

**Keywords:** NK cell expansion, feeder-free, nanoparticle, adoptive cell therapy, allogenic cell therapy

## Abstract

Adoptive cell therapy has progressed rapidly following the success of CAR-T therapy, with NK cells emerging as strong candidates for next-generation treatments. NK cells naturally recognize abnormal cells without genetic modification and do not cause graft-versus-host disease, making them suitable for mass production from donor blood. Traditional NK cell expansion often relies on cancer-derived feeder cells, posing safety and quality concerns. This review examines both feeder cell-based and feeder-free NK cell culture methods. We analyze the limitations of feeder-based approaches and categorize feeder-free strategies, including cytokine combinations, antibody stimulation, blood components, nanoparticles, and hydrogels. Among these, nanoparticle-based methods show strong potential for enabling safe, scalable, and standardized NK cell expansion without the risks associated with feeder cells. In this paper, we review recent advancements in NK cell culture techniques and propose the potential of nanoparticle technology in developing feeder-free NK cell culture methods.

## 1 Introduction

Cancer immunotherapy, represented by immune checkpoint inhibitors (ICIs), has revolutionized cancer treatment by showing the potential for durable responses with relatively low toxicity (1). ICIs led some patients to complete remission, triggering diverse research about the curability of cancer through immune-based approaches. However, most patients fail to achieve long-term remission, prompting intense research into predictive biomarkers and combination strategies (2, 3). In this context, chimeric antigen receptor (CAR) T cell therapy has emerged as a breakthrough, particularly in hematological malignancies, demonstrating outstanding efficacy in patients refractory to conventional treatments (4). The therapeutic concept of expanding and infusing tumor-targeting cytotoxic lymphocytes has validated the potential of gene and cell therapies. Despite initial concerns about cytokine release syndrome and neurotoxicity, improved clinical management has rendered these adverse effects more controllable, shifting the focus toward broader accessibility and standardization of CAR therapies (5).

A major limitation of current CAR-T cell therapy lies in its autologous nature, requiring personalized manufacturing from patient-derived T cells (6). This process is time-consuming and often suboptimal in patients with advanced disease. As a solution, allogeneic cell therapy—leveraging immune cells from healthy donors—has gained attention as a promising strategy for off-the-shelf cellular products (7). Among candidate effector cells for allogeneic therapy, natural killer (NK) cells have advantages. Unlike T cells, NK cells do not induce graft-versus-host disease (GvHD) and are abundantly present in peripheral blood, enabling scalable isolation (8). Numerous studies have explored NK cells as next-generation therapeutic platforms, and recent clinical data have shown that CAR-NK cells can achieve antitumor efficacy comparable to CAR-T cells, highlighting the translational potential of NK cell-based immunotherapy (9).

A critical requirement for the clinical success of NK cell therapy is the development of efficient and safe expansion methods. Enhancing the fold expansion of NK cells not only improves therapeutic efficacy but also contributes to cost reduction and quality assurance (10). Compared with T cells, the ex vivo expansion of NK cells is generally more challenging. While T cells can be robustly activated and expanded using CD3/CD28 stimulation alone (11), NK cell activation requires the integration of multiple activating and inhibitory signals (12). This complexity makes it difficult to identify a single molecular combination equivalent to CD3/CD28 for NK cells. Consequently, substantial efforts continue to be directed toward the development of advanced NK cell culture methods that can achieve robust expansion while meeting the requirements for clinical applicability (13). This review summarizes the current advances in feeder-free NK cell expansion technologies and discusses future directions toward standardized NK cell manufacturing for adoptive immunotherapy. Since the experimental conditions and methods of data presentation vary across studies, direct comparison is challenging. However, we summarized the reported results of NK cell expansion focusing on fold expansion, NK cell purity, and clinical compatibility, which is able to help in understanding the current trends in NK cell culture strategies. To this end, various strategies have been proposed to optimize NK cell culture systems.

## 2 Feeder cell-based NK cell expansion

**Figure 1:**
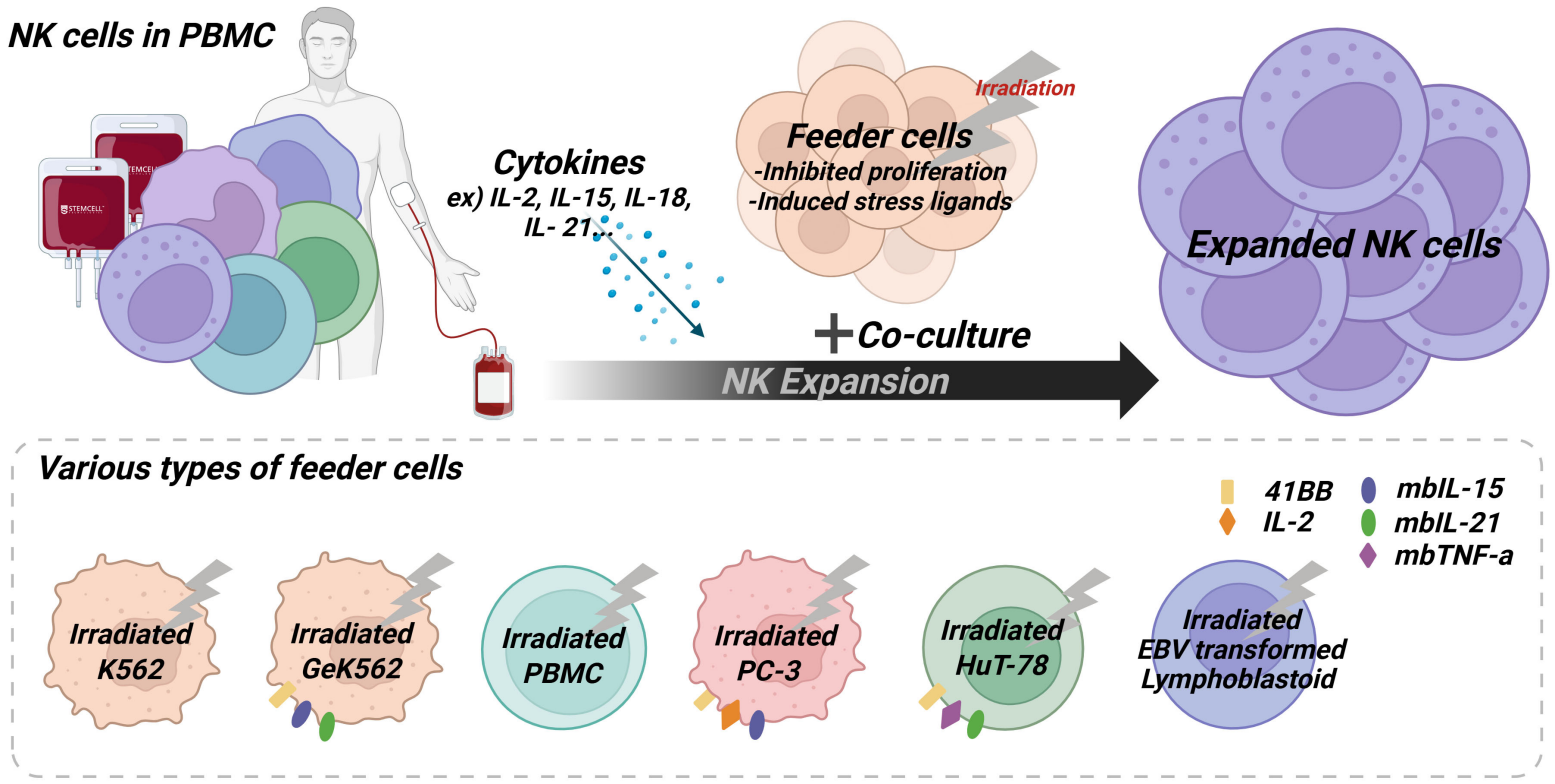
The feeder cell-based culture method for anticancer treatment using human blood-derived NK cells. Feeder cells, including cancer cells, PBMCs, and EBV-transformed lymphoblastoid cells, are treated with γ-ray irradiation to inhibit proliferation and induce the expression of stress ligands. Genetically engineered feeder cells expressing NK cell–stimulatory molecules have also been developed and are widely used to enhance expansion efficiency. Expanded NK cells can be obtained by co-culturing blood-derived mononuclear cells with γ-irradiated feeder cells. Created with BioRender.com.

Feeder cells for NK cell expansion are mostly treated with γ-ray irradiation to inhibit their proliferation and induce them to express stress ligands that can help to stimulate NK cells (**Figure 1**) (14). Source of feeder cells are diverse including PBMCs, tumor cell lines and EBV-transformed lymphoblastoid cells. The process of utilizing PBMC as feeder cells involves isolating NK cells from PBMC and subsequently subjecting the remaining non-NK cells to γ-ray irradiation for utilization. This approach, utilizing normal cells from the bloodstream as feeder cells, offers enhanced stability compared to employing cancer cell lines (15, 16). NK cell expansions using γ-ray irradiated PBMCs show fold expansion ranging from 794 ± 115.6 (15) on day 21 and from 80–419 on day 12 (16). K562 cells are the most widely used feeder cell for NK cell expansion and diverse genetically engineered K562 (GeK562) cells have been developed to improve NK cell expansion by expressing ligands that can help. GeK562 cells expressing 4-1BB ligand, membrane-bound IL-15 (mbIL-15) and membrane-bound IL-21 (mbIL-21) showed superior NK cell expansion as used feeder cells rather than K562 cells [fold expansion: 842-fold/purity: 91.5%/clinical suitability: not mentioned] (17). Additionally, GeK562 cells expressing molecules such as OX40 ligand [fold expansion: 578.9-2,959.5 fold/purity: 90%/clinical suitability: not mentioned] (18), membrane-bound IL-18 [fold expansion: 9,860-fold/purity: ≥98%/clinical suitability: not mentioned] (19, 20), and HLA-E [fold expansion: 1,632.43–12,748 fold/purity: 80%/clinical suitability: not mentioned] (21, 22) have been developed. Various GeK562 cells have been developed, and although there are differences in NK cell expansion observed in each study, it is difficult to determine which GeK562 is better based on fold expansion values. This difficulty arises not only from differences in feeder cells but also from variations in experimental conditions such as culture medium (23).

Other tumor cell lines have been developed as feeder cells by genetic engineering for effective NK cell stimulation. PC-3 cell-line (prostate cancer) derived feeder cells having genetic engineering to express prostate stem cell antigen (PSCA) with IL-2, 4-1BBL and mbIL-15-mutDAP12 fusion protein on the cell membrane. An example of this is PC3^PSCA^ -derived feeder cells, which induced the expression of high affinity IL-2 receptor through upregulation of DNAM-1 and NKG2C, resulting in fold expansion values of 86.2 ± 38.8 for PC3^PSCA^ -IL-2-4-1BBL-mIL15d (24). Lymphoblastoid cell-lines transformed by Epstein–Barr virus (EBV) infection was used as feeder cells for NK cell expansion and showed 490 ± 260-fold expansion on day 21 with enhanced cytotoxicity functions (25, 26). HuT 78 cell-line (cutaneous T cell lymphoma) was systemically investigated to maximize the NK cell fold expansion and an engineered form of feeder cells to express 4-1BB ligand, membrane-bound TNF-α and mbIL-21 showed over 2,000-fold expansion on day 21 (27). Although early studies on genetically engineered feeder cells investigated the co-expression of mbIL-15 and mbIL-21, current feeder-based NK cell expansion most commonly employs soluble IL-15 supplementation with feeder cells expressing mbIL-21. IL-15 is required continuously to sustain NK cell proliferation, but feeder cells are rapidly cleared within a few days of culture, meaning mbIL-15 expression is less effective. In contrast, IL-21 primarily acts in the initial stage of NK cell culture, and its early signaling has been shown to exert long-lasting effects throughout the entire expansion process (18).

The feeder cell-based NK cell expansion is the most widely used methods for NK cell studies including clinical trials due to its high fold expansion rates. However, this method is largely affected by the feeder cell quality thus, culture or storage conditions of the feeder cells should be tightly managed. For this reason, development of new NK cell expansion methods that can expand NK cells and be stored easily is required to the quality control of expanded NK cells for clinical use.

## 3 Feeder-free NK cell expansion

### 3.1 Cytokine combination

Diverse investigations have been conducted to expand NK cells without using feeder cells, and the most fundamental approach was to identify the optimal cytokine combination. Depending on the treatment schedule, concentration, and combination, cytokines differently effect on NK cell expansion and functions. Purified NK cells cultured with treatment of IL-15 alone showed a significant increase in NK cell numbers during the initial 10 days, (all protocol with IL-15) whereas treatment with IL-21 alone failed to induce NK cell proliferation. NK cell culture using IL-15 treatment showed enhanced fold expansion under temporal IL-21 treatment at the beginning of NK cell culture (2- and 10- fold), it means that IL-21 alone is not sufficient to induce NK cell proliferation but can support to NK cell proliferation with treating other cytokines (28). Other cytokine combination including IL-2, IL-18, rIL-27 showed improved NK cell fold expansion (17.19 ± 4.85-fold) (29). Basically, IL-2 and IL-15 are essential cytokines showing mild NK cell proliferation when treated alone. IL-18, IL-21 and IL-27 are supported cytokines showing less NK cell proliferation when treated alone however, enhanced NK cell fold expansion when combined with essential cytokines. NK cells cultured with combination of essential with supported cytokines also showed increased expression of activation receptors and enhanced functions such as degranulation of cytotoxic vesicles and IFN-ɣ secretion under target cell stimulation.

### 3.2 Antibody stimulation

In T cell culture, effective proliferation can be induced using stimulation with anti-CD3/28 antibodies. Similarly, various studies have been conducted in NK cells to induce proliferation without feeder cells by utilizing combinations of agonist antibodies. Interestingly, the anti-CD3 agonist antibody clone OKT-3, commonly used for T cell proliferation, also exhibits efficacy in NK cell proliferation. This effect is attributed to the cytokines secreted by T cells in response to OKT-3 stimulation, which facilitate NK cell proliferation. NK cells cultured using OKT-3 stimulation from PBMC showed 700-fold expansion and 55% of NK cell purity (CD3^-^/CD56^+^) meaning OKT-3 alone is not ideal for replacing feeder cell-based NK cell culture (30). Antibody combination using minimal dose of OKT-3 (0.001 ~ 0.01 µg/mL) and 20 µg/mL anti-CD52 antibody showed improved NK cell expansion with around 60% of NK cell purity [fold expansion: ~1,000-fold/purity: ~60%/clinical suitability: possible] (31). Another antibody combination using OKT-3 with anti-CD16 agonistic monoclonal antibody increase the NK cell purity to 75.2 ± 16.3%, while T cells (CD3^+^/CD56^-^) decreased from 66.5 ± 7.8% to 16.5 ± 6.2% after expansion [fold expansion: 352 ± 344-fold/purity: 75.2 ± 16.3%/clinical suitability: possible] (32). However, anti-CD16 stimulation showed wide range of donor variations (NK cell purity range 33.9 ~ 93.2%) and the variations was maintained regardless of anti-CD16 antibody clones (33). NK cell culture methods using OKT-3 have limited NK cell purity due to residual T cells, thus antibody combinations excluding OKT-3 have been studied for NK cell culture from T cell-depleted PBMCs. Stimulation of T cell-depleted PBMCs with anti-NKp46 and anti-CD16 antibody combination resulted in over 98% of NK cell purity with 1,000-fold expansion (34). Both NK cell purity and fold expansion are superior compared to other antibody combinations, but differences in culture reagents, such as the type of culture media and cytokine combinations, also influence the results.

### 3.3 Human blood derived components

The use of serum, human serum (HS), or fetal bovine serum (FBS), in cell culture carries the risk of allergic reactions, and its composition is not well characterized, making it difficult to maintain consistency in culture conditions and performance during manufacturing processes. Autologous plasma as a culture supplement is best to Xeno-free culture media and it showed prominent NK cell expansion [fold expansion: 496.5 ± 55.0-fold/purity: 99.0 ± 0.6%/clinical suitability: possible] (35). However, securing enough amount of plasma does not come easy thus, investigation of replacement for the serum component is needed. In the study of stem cell-derived NK cell culture, human platelet lysate (hPL) was used instead of human serum. After NK cell differentiation and expansion from CD34^+^ stem cells in hPL- or HS-containing media, both media were capable of producing mature NK cells. However, hPL was found to promote greater NK cell proliferation and expression of activation receptors, such as CD16 and NKp46, indicating that hPL may aid in the generation of mature and cytotoxic NK cells (36). In other study showed that hPL containing media with Cloudz which is a stimulator of NK cells decorated with anti-CD2 and anti-NKp46 agonist antibodies can be used for NK cell expansion from PBMCs [fold expansion: 387 ± 100-fold/purity: 71% ± 15%/clinical suitability: possible] (37). These results mean that serum contained in NK cell culture media can be replaced to hPL for the Xeno-free protocol of NK cell expansion.

### 3.4 Nanoparticle

**Figure 2:**
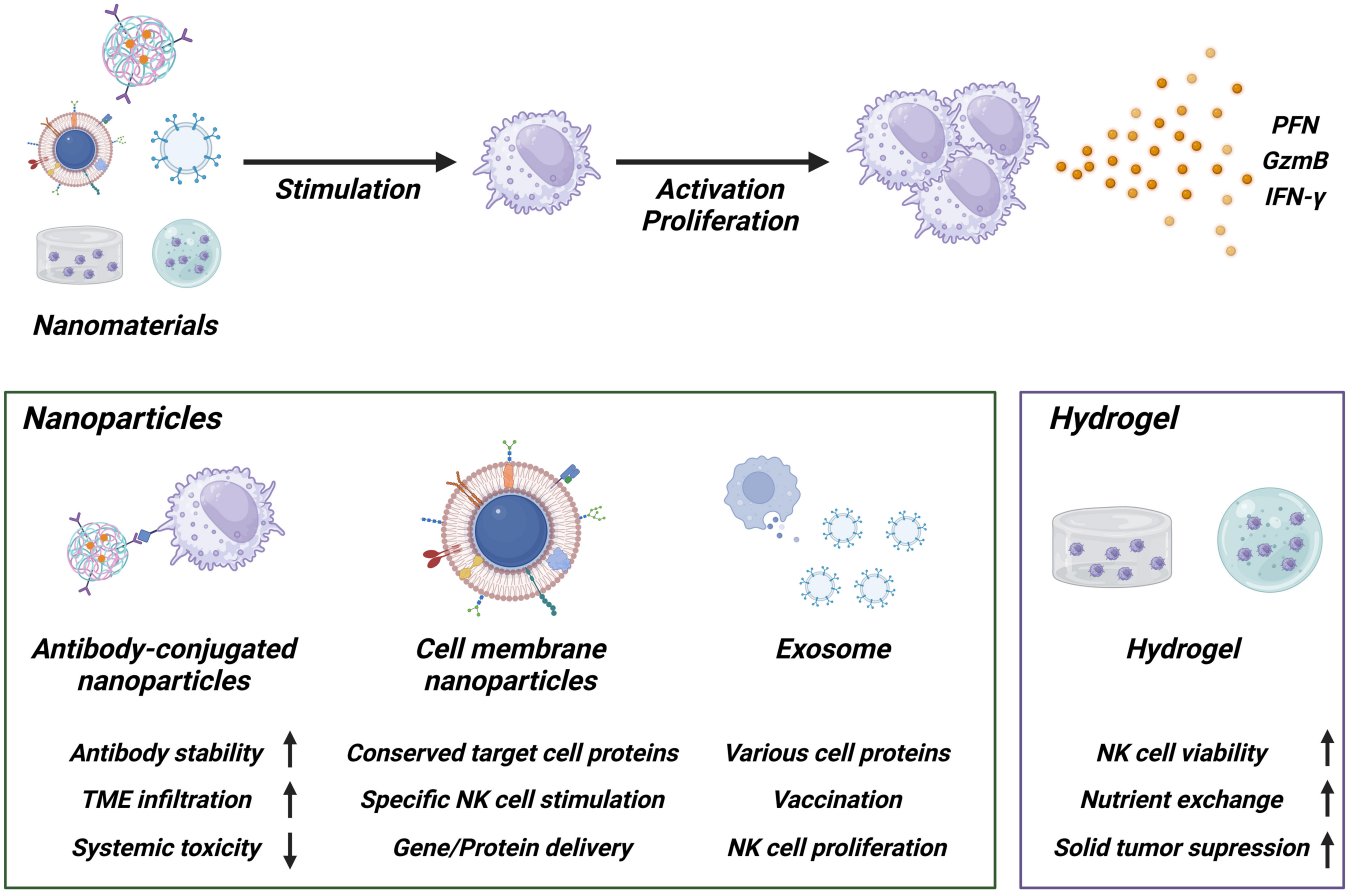
Schematic of NK cell activation using nanomaterials. Antibody-conjugated nanoparticles enhance the stability of antibodies, improve tumor microenvironment (TME) penetration, and reduce systemic toxicity. Cell membrane nanoparticles and exosomes maintain the protein expression profile of the originating cells, demonstrating high functionality and suitability for gene or protein delivery. Hydrogels help maintain NK cell functionality and survival within the TME condition, enhancing therapeutic efficacy against solid tumor. Created with BioRender.com.

Nanomaterials are substances having nano sizes ranging from 1 nm to 200 nm, and they have been widely investigated for various research such as drug and gene delivery (38). Nanomaterials possess own unique properties following 1) the ability to have multivalent molecules on their surface, 2) the ease of modifying their functionality through chemical conjugation, 3) effectively uptake into targeted cells through the receptor-mediated endocytosis pathway (38–40). Recently, nanomaterial usages for NK cell expansion have been investigated to utilize their unique advantages (**Figure 2**).

One of the strategies is that fabrication of protein-conjugated nanogels. For instance, nanogels incorporating IL-21 and CD45 (ILNPs) were developed and shown to “hitchhike” NK cell. Incorporation of IL-21 allowed controlled activation in the tumor microenvironment, thereby reducing systemic toxicity and inducing IL-12-mediated innate immune activation (41). Beyond the chemical conjugation methods, NK cell expansion with cell membrane-derived nanomaterial has been attracted as next generation methods because it contains whole membrane protein profiles that are consistent with profiles of source cells, supporting recognition of target cell proteins (42, 43). The Park group developed cancer cell membrane-expressed magnetic nanoparticles that specifically stimulate NK cells and also functioned as gene delivery carriers to induce EGFR-CAR expression. Multifunctional nanoparticles (MF-NPs) coated with caffeic acid, polydopamine, and polyethyleneimine show reduced toxicity once they are effectively internalized into NK cells. They then induce the expression of EGFR-CAR on the NK cell surface due to their gene delivery carrier ability (44).

Feeder cell-mimicking strategies have also been reported. The Copik group demonstrated that feeder cell membrane-derived particles can be used to replace common feeder cell-based NK cell expansion methods. When plasma membrane particles (PM-particles) were used in place of live feeder cells, the results showed that NK cell expansion and NK cell phenotype were comparable (45). Similarly, the Wang group showed another application for nanomaterials-based NK cell expansion by developing cell membrane-encapsulated magnetic nanoparticles for activating NK cells and enhancing their antitumoral efficacy (46). Extracellular vesicles (EVs), another type of nanomaterial secreted from cells, contain various specific proteins from the original cells and have been investigated for use in vaccines, immune regulation, and other applications (40, 47). The Chaput group demonstrated that dendritic cell-derived exosomes (Dex) possessing functional MHC/peptide complexes act as promoters of T cell-dependent tumor rejection and supported NK cell proliferation and activation via IL-15Rα and NKG2D signaling (48).

Taken together, nanomaterials represent customizable feeder-free platforms that mimic cell-to-cell interactions and can enhance NK cell expansion while reducing toxicity. In addition, because they can be produced in a well-defined manner and stored long-term under GMP-compatible conditions, they hold promise for clinical translation, although further evaluation of safety, reproducibility, and cost is required.

### 3.5 Hydrogel

Hydrogel-based adoptive cell therapy using NK cells has also been explored, particularly against solid tumors. Hydrogels provide three-dimensional, biocompatible scaffolds that can encapsulate the immune cells with supportive factors, thereby preserving their viability and anti-tumor activity in the immunosuppressive tumor microenvironment (TME). While many studies have employed NK-92 cells as a model system, these approaches are broadly applicable to NK cell-based therapies.

For example, NK-92 cells encapsulated in sodium alginate/gelatin hydrogels (ALG/GEL-2) showed higher viability and cytotoxic function compared with cells cultured without hydrogels (49). Microspheres fabricated using PEO/ALG showed high permeability and efficient nutrient exchange, enabling NK-92MI cell survival rates of over 85% after 72 hours of culture, with viability maintained for up to 14 days. Encapsulated NK-92MI cells effectively destroyed tumor cells through secretion of perforin and granzyme and porous hydrogel microspheres further protected NK-92MI cells from the immune rejection response, supporting their survival and proliferation (50). Another study created NK-CellDex by encapsulating NK-92 cells engineered with CellDex into GelMA bioink-based hydrogels. These hydrogel capsules supported cell proliferation, increased viability, and maintained cell morphology similar to 2D culture, indicating that hydrogels can support NK cell expansion even after encapsulation (51).

Mechanistically, hydrogels help protect NK cells from TME-derived inhibitory signals, while supporting sustained nutrient exchange. They can also be engineered to deliver stimulatory molecules that enhance NK cell activity. Importantly, beyond serving as delivery vehicles, hydrogels provide a microenvironment that may promote NK cell proliferation *in vivo*, thereby offering the potential for feeder-free expansion strategies. Moreover, localized and sustained NK cell delivery could reduce systemic toxicity and enhance therapeutic efficacy.

Overall, hydrogel-based NK cell delivery systems represent promising and translatable feeder-free approaches. They offer advantages such as biocompatibility, modular design, and scalability, while also holding potential to contribute to NK cell expansion. Nonetheless, challenges such as regulatory approval and reproducibility remain to be addressed before clinical applications.

## 4 Conclusions

In summary, feeder-free strategies using cytokines, antibodies, and blood-derived components have advanced NK cell expansion but remain limited by inconsistent proliferation and reproducibility. Although defined cytokine- or antibody-based systems would be more suitable for clinical use, their development is made difficult by the complex signaling requirements of NK cells. As a result, feeder cell–based systems are still widely used, though their clinical translation raises safety and standardization concerns. Emerging nanomaterial- and hydrogel-based platforms provide GMP-compatible, storable alternatives that can deliver stimulatory cues, protect cells from immunosuppression, and sustain viability. These approaches represent new possibilities for overcoming current limitations and advancing NK cell therapies.
